# To William Sandwith, Esq.

**Published:** 1803-06-01

**Authors:** T. Christie

**Affiliations:** Columbo


To WILLIAM SANDWITH, Esq.
*Secretary to the Medical Board at Bombay.*

Sir,

I Have sincere pleasure in acquainting you, for the information of the Medical Board at Bombay, that after various failures at the different stations on this island, the Vaccine Inoculation has at length happily succeeded at Trincomallie, with matter taken from the arm of a child of European parents at Bombay, on the eighth day from inoculation, being the 10th of July.

This matter was sent by Mr. Helenas Scott, under cover to Mr. North, and did not reach Trincomallie till the 11th of August; several patients were on that day inoculated with this matter by Mr. Rogers, Medical Superintendent at Trincomallie; but, as in former cases, it failed of success in all, except in John Sybelle, a half-cast boy of twelve years of age, in whom it very fortunately took effect. On the 20th of August, being the ninth day from inoculation, a distinct pustule was formed on the inoculated part, bearing the characteristic marks of the Cow-pox, and attended with pain in the axilla, and slight fever, so that Mr. Gilbert Hall, Surgeon of the Malay Corps, who, in consequence of the absence of Mr. Rogers, had charge of the patient, had no hesitation in pronouncing it to be the true Vaccine disease. He immediately inoculated thirteen patients from the arm of this boy, in all of whom he informs me the inoculation had evidently taken effect on the 26th of August. The most effectual measures have been ordered by this government, for keeping the contagion, and extending it as expeditiously as possible to the different stations on this island, and the Coromandel Coast, by means of inoculated patients. The fortunate event of John Sybelle's inoculation with matter thirty-two days old, should teach us not to despair of success with dried matter, which has even been kept a considerable time in a hot climate; but the very frequent failures with dried matter, both in Europe and India, shew that it is at best but a very uncertain means of conveying the contagion to any considerable distance. I beg leave to congratulate the gentlemen of the Bombay Medical Board, on the success of their endeavours in the cause of humanity, and to return them my thanks for the benefits they have conferred on this settlement, by introducing the Vaccine disease into India, and extending it to Ceylon.

I remain, &c.

*Columbo,*
*September* 1, 1802.

T. CHRISTIE.

